# A case report dysregulated neutrophil extracellular traps in a patient with propylthiouracil-induced anti-neutrophil cytoplasmic antibody-associated vasculitis

**DOI:** 10.1097/MD.0000000000015328

**Published:** 2019-04-26

**Authors:** Kanako Watanabe-Kusunoki, Nobuya Abe, Daigo Nakazawa, Kohei Karino, Fumihiko Hattanda, Yuichiro Fujieda, Saori Nishio, Shinsuke Yasuda, Akihiro Ishizu, Tatsuya Atsumi

**Affiliations:** a Department of Rheumatology, Nephrology and Endocrinology, Faculty of Medicine and Graduate School of Medicine; b Faculty of Health Sciences, Hokkaido University, Sapporo, Japan.

**Keywords:** ANCA, drug-induced vasculits, NETs

## Abstract

**Rationale:**

Neutrophil extracellular traps (NETs) are immune defence systems that release extracellular chromatin and myeloid granules including myeloperoxidase (MPO) to kill pathogens. An experimental animal study recently demonstrated that disordered NETs induced by propylthiouracil (PTU) could contribute to the production of MPO anti-neutrophil cytoplasmic antibody (ANCA) and the development of ANCA-associated vasculitis (AAV). However, the role of dysregulated NETs in the pathogenesis of human AAV remains unclear.

**Patient concerns:**

We report a 19-year-old woman with Graves’ disease on PTU presented fever, polyarthralgia, and lung hemorrhage with high titer of MPO-ANCA. This patient had a variety of atypical ANCAs and disordered NETs *in vitro*.

**Diagnoses:**

A diagnosis of PTU-induced AAV (PTU-AAV).

**Interventions:**

The PTU was discontinued and she was treated with immunosuppressants and plasmapheresis for reducing pathogenic autoantibodies.

**Outcomes:**

Clinical manifestations including fever, polyarthralgia, and lung hemorrhage were on remission with a decrease of dysregulated NETs.

**Lessons:**

The clinical course of this PTU-AAV case indicated that dysregulated NETs would play a role in the development of ANCA and the pathogenesis of AAV.

## 1 Introduction

Anti-neutrophil cytoplasmic antibody (ANCA)-associated vasculitis (AAV) is a necrotizing vasculitis that predominantly affects small vessels.^[1,2]^ Although its pathogenesis is unclear, neutrophil extracellular traps (NETs) are assumed to contribute to the development of AAV. NETs release extracellular chromatin with histones and myeloperoxidase (MPO) to trap and kill microbes, ultimately being properly digested.^[3,4]^ In patients with MPO-AAV, soluble NETs in blood and focal NETs in the lesions of crescentic glomerulonephritis were detected.^[5]^ These findings suggest that NET formation triggers an autoimmune response, resulting in ANCA production and the development of vasculitis.

Propylthiouracil (PTU), a drug that commonly causes ANCA seropositivity,^[6,7]^ can also cause vasculitis.^[8,9]^ We^[10]^ previously showed that PTU-treated neutrophils revealed a morphological abnormality during NET formation in that they were barely degraded by DNase I. Furthermore, a study of PTU-treated rats indicated that dysregulated NETs could lead to ANCA production and the subsequent development of pulmonary capillaritis and glomerulonephritis.

We hypothesized that the neutrophils of a patient with PTU-induced AAV (PTU-AAV) become dysregulated NETs, which cause the development of pathogenic ANCA and vasculitis.

## 2 Methods

### 2.1 Samples

Blood samples were obtained from patient with PTU-AAV, patients with idiopathic microscopic polyangiitis (MPA), and healthy controls (HC). This study was approved by the Institutional Review Board of Hokkaido University Hospital. We obtained written informed consent from all participants. Neutrophils isolated by polymorphprep (Axis-Shield) were suspended in Roswell Park Memorial Institute (RPMI) medium (1 ×10^6^ cells/mL) and seeded into 8-well microslides or 24-well plates in a 5% carbon dioxide atmosphere at 37°C for 30 minutes prior to the stimulation. The patient and HC serum samples were stored at −80°C until use and immunoglobulin G (IgG) was eluted from the serum using a protein G SpinTrap column (GE Healthcare).

### 2.2 NET regulation in patient neutrophils

After preincubation, patient and HC neutrophils were cultured with or without 50 nM phorbol myristate acetate (PMA) for 3 hours at 37°C. After culturing, the neutrophils were incubated with DNase I (1 U/mL; Roche) for 15 minutes. After fixation in 4% paraformaldehyde, specimens were made to react with rabbit anti-hMPO antibody (5 μg/mL; Abcam) for 24 hours at 4°C and then reacted with a 1:500 dilution of Alexa Fluor 594-conjugated goat anti-rabbit IgG (Invitrogen) for 60 minutes at room temperature. The DNA was stained with DAPI contained in the mounting solution (Sigma-Aldrich).

### 2.3 Analysis of histone citrullination in neutrophil under natural conditions

Neutrophils from HC and patient (active and remission phase) were prepared for sodium dodecyl sulphate–polyacrylamide gel electrophoresis by sonication in RIPA buffer (Thermo Fisher Scientific, Waltham, MA). For evaluation of the NET signalling pathway, histone 3 (H3) citrullination was examined by immunoblotting using anti-Cit H3 antibody (Abcam) and quantified using Image J software. Actin was used as an internal control. Healthy neutrophils treated with 50 nM PMA served as the positive control.

### 2.4 NET induction ability of patient ANCA

Neutrophils from a healthy donner (1 × 10^6^/mL) were primed with tumor necrosis factor (TNF)-α (5 ng/mL) and incubated with purified IgG (400 μg/mL) obtained from patient with PTU-AAV, patients with idiopathic MPA, and HC. After 3 hours of incubation at 37°C, NET induction was assessed with LDH assay as previously reported.^[11]^

## 3 Case report

**Figure 1:**
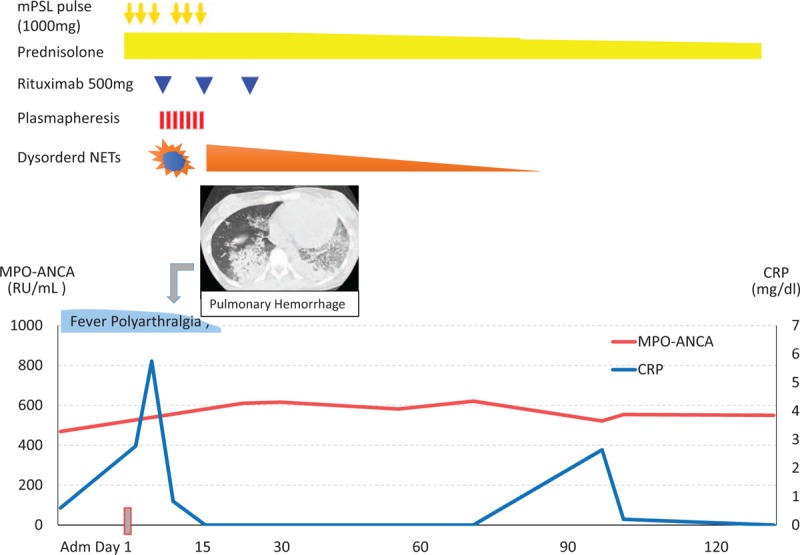
Clinical course of PTU-AAV patient. AAV = ANCA-associated vasculitis, mPSL = methylprednisolone, PTU = propylthiouracil.

A 19-year-old woman with a 1-month history of a high fever up to 40°C and polyarthralgia was admitted to our unit. Two years prior to this admission, she was diagnosed with Graves’ disease and achieved euthyroid status with PTU treatment. On admission, pain and swelling of the right forearm was noted without a skin rash. Blood test results showed elevated levels of C-reactive protein, MPO-ANCA (469 RU/mL; normal range, <2.0 RU/mL), antinuclear antibody, anti-double-stranded DNA antibody, anticardiolipin antibody, and lupus anticoagulants with a biological false-positive reaction. Results of urinalyses and renal function analyses were normal. Thus, she was diagnosed with PTU-AAV, thus the PTU was discontinued. Since her symptoms did not improve, methylprednisolone pulse therapy was added (1000 mg daily for 3 consecutive days), followed by prednisolone (50 mg daily). However, she then developed a diffuse alveolar hemorrhage (Fig. 1). We administered additional 2 courses of methylprednisolone pulse therapy, rituximab (375 mg/m^2^ once weekly), and plasmapheresis. With those intensive immunosuppressive treatments, her clinical manifestations were on remission during a 1-year follow-up.

### 3.1 Results of NETs regulation

**Figure 2:**
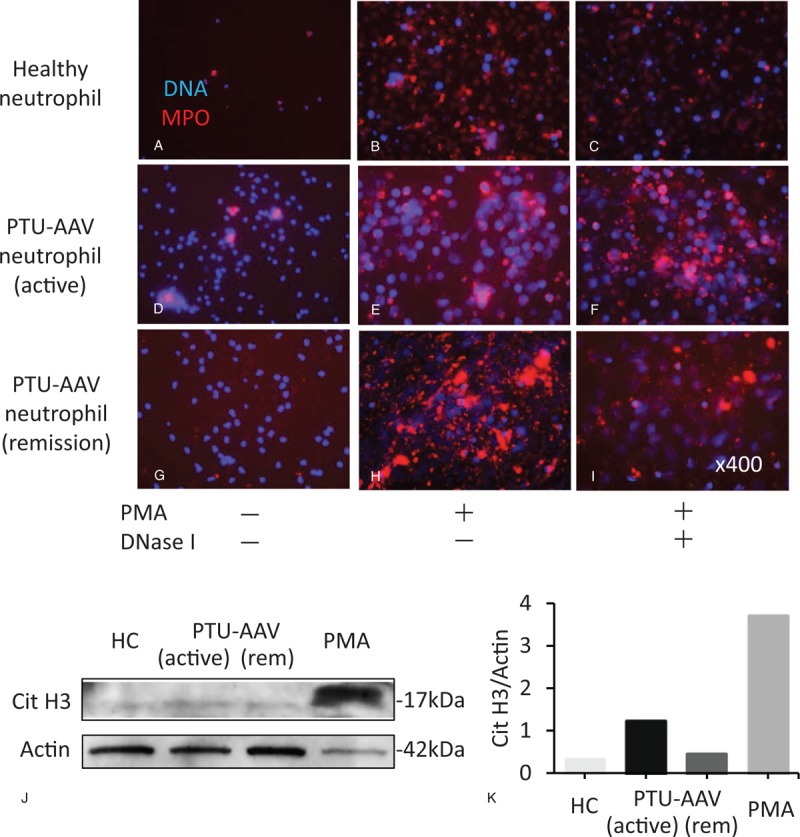
The presence of excessive and disordered NETs. Immunofluorescent images of neutrophil extracellular traps (NETs) of healthy control (A–C), a patient with propylthiouracil (PTU)-induced ANCA-associated vasculitis (AAV) in the active (D–F) and remission phase (G–I). Red indicates myeloperoxidase (MPO) and blue indicates DNA. (A, D, and G) Un-stimulated neutrophils. (B, E, and H) Neutrophils stimulated with phorbol myristate acetate (PMA). (C, F and I) PMA-induced NETs after DNase I treatment. Original magnification is ×400 in (A–I. J) The expression of histone citrullination (Cit H3) in healthy control and PTU-AAV neutrophils (active and remission phase) was detected by immunoblotting with actin as a loading control. As a positive control of Cit H3 expression, neutrophils were treated with 50 nM PMA. (K) Quantitative analysis of Cit H3 in immunoblotting is performed by ImageJ software. AAV = ANCA-associated vasculitis, ANCA = anti-neutrophil cytoplasmic antibody, MPO = myeloperoxidase, PMA = phorbol myristate acetate, PTU = propylthiouracil.

We first examined NET regulation in the neutrophils of a patient with PTU-AAV. The immunofluorescent images and immunoblotting showed NET formation with hyper-citrullinated histone in PTU-AAV neutrophils during the active phase (Fig. 2D, J, K). These neutrophils after PMA stimulation were induced to form massive NETs, which were not degraded by DNase I (Fig. 2E and F). In contrast, HC and PTU-AAV remission phase neutrophils did not show excessive NET induction (Fig. 2A, G, J, K) and these NETs were degraded by DNase I (Fig. 2B, C, H, I).

**Figure 3:**
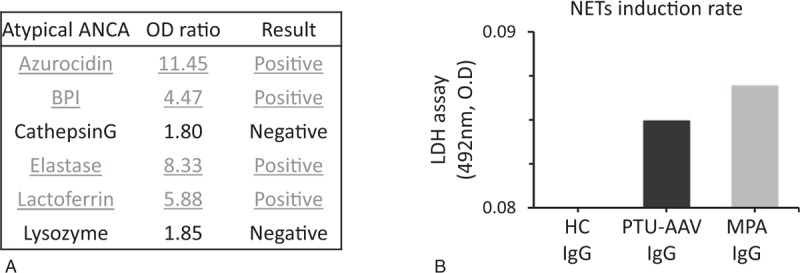
Atypical ANCA and ANCA's NET inducibility. (A) Atypical ANCAs were detected by ANCA panel kit (enzyme-linked immunosorbent assays). (B) Lactate dehydrogenase (LDH) release from neutrophils treated with purified immunoglobulin G obtained from individuals with PTU-induced AAV and microscopic polyangiitis (MPA) and a healthy donor were assessed as NETs inducibility. AAV = ANCA-associated vasculitis, ANCA = anti-neutrophil cytoplasmic antibody, PTU = propylthiouracil.

Next, we hypothesized that disordered NETs could play a role as auto-antigens. To examine the presence of autoantibodies to NET components, we tested atypical ANCA using an ANCA panel kit (enzyme-linked immunosorbent assays, Euro Diagnostica, Sweden). Indirect immunofluorescence of the neutrophils revealed a perinuclear-ANCA staining pattern, which reacted to azurocidin, bactericidal/permeability increasing protein, elastase, and lactoferrin (Fig. 3A). Furthermore, to examine the property of these ANCA to induce NETs, healthy neutrophils primed with TNF-α (5 μg/mL) were incubated with PTU-AAV IgG (ANCA), HC IgG, and idiopathic MPA IgG (MPO-ANCA IgG). The LDH assay indicated that PTU-AAV IgG and MPA-IgG had a high NETs-inducibility compared to HC-IgGs (Fig. 3B).

## 4 Discussion

We hypothesized that disordered NET regulation in patients with PTU-AAV could contribute to the development of ANCA and vasculitis. Our data confirmed this concept and indicated that PTU-induced DNase I-resistant NET could play a role as auto-antigens leading to the production of various ANCA as anti-NET antibodies, accelerating further NET formation. Although NETs are composed of innate immune system substances, excessive NETs or diminished NET clearance is involved in the risk of autoreactivity to NET components and the onset of autoimmune disease.^[12]^ Hakkim et al^[13]^ showed that patients with systemic lupus nephritis have prolonged NET formation due to an impaired DNase regulation, indicating the involvement of NETs in autoimmunity. Kessenbrock et al^[5]^ first reported the relevance of NETs in ANCA vasculitis. ANCA-stimulated neutrophils undergo NET formation and the NETs were identified in crescentic glomerulonephritis in AAV-patients. An experimental animal study showed that PTU-treated rats have abnormal conformations and impaired NET degradation involved in the pathogenesis of MPO-ANCA generation and AAV. NETs in PTU-treated neutrophils reportedly have DNase I resistance, leading to auto-reaction to NET components.^[10]^

Here we demonstrated that PTU-AAV patient's neutrophils showed massive NETs under un-stimulated and stimulated states. These NETs in PTU-AAV were not degraded by DNase I as reported in a previous study^[10]^ in which PTU was added to healthy neutrophils in vitro. Therefore, NETs status in this case report would represent our previous in vitro observation. In addition, we confirmed the presence of hyper-citrullinated histones in PTU-AAV neutrophils, indicating the activation of NET signalling pathway. Serum levels of cell-free DNA were higher in the patient with PTU-AAV (data not shown), presumably corresponding to increased circulating NETs. In turn, various kinds of antibodies to NET components were detected in this patient as atypical ANCA. PTU is known to bind to MPO and alter the haem iron structure periphery, resulting in neo-antigen production.^[14]^ However, these data from the patient and a previous animal study suggest that exposure to dysregulated NETs by PTU could potentially lead to a breakdown in immunological tolerance resulting in the development of anti-NET antibodies. Furthermore, PTU-AAV IgG including MPO-ANCA and atypical ANCA primes neutrophils to undergo NET formation. This is the first case report to demonstrate that abnormal and dysregulated NETs persisted in the patient with PTU-AAV and various ANCA. The vicious cycle of NET-ANCA via PTU seems to play a significant role in the development of vasculitis.

We acknowledge that this study has several limitations. The data were obtained from only one patient and may not reflect the general population of patients with PTU-AAV. Therefore, our findings should be confirmed by other series of the patients. Second, we could not show direct evidence of the pathophysiology of the dysregulated NETs in the development of vasculitis syndrome in this patient. However, we believe that useful information regarding the interaction between impaired NETs and AAV progression may be provided by further studies.

In this study, we demonstrated the aggregation of abnormal and dysregulated NETs in a patient with PTU-AAV. The relevance between impaired NET regulation and subsequent ANCA production is closely involved with the pathogenesis of AAV.

## Author contributions

NA, KK, YF, SY, and TA cared for the patient. KW, DN, YF FH, AI and SN contributed to the conception and design of the work, and analysis and interpretation of data. All authors contributed to writing the report.

**Conceptualization:** Nobuya Abe, Daigo Nakazawa, Yuichiro Fujieda, Saori Nishio, Shinsuke Yasuda, Akihiro Ishizu, Tatsuya Atsumi.

**Investigation:** Kanako Watanabe-Kusunoki, Fumihiko Hattanda.

**Resources:** Kohei Karino.

**Writing – original draft:** Kanako Watanabe-Kusunoki, Nobuya Abe.

**Writing – review & editing:** Daigo Nakazawa, Tatsuya Atsumi.
